# Scientists’ opinions and attitudes towards citizens’ understanding of science and their role in public engagement activities

**DOI:** 10.1371/journal.pone.0224262

**Published:** 2019-11-13

**Authors:** Carolina Llorente, Gema Revuelta, Mar Carrió, Miquel Porta

**Affiliations:** 1 Studies Centre on Science, Communication and Society, Department of Experimental and Health Sciences, Universitat Pompeu Fabra, Barcelona, Spain; 2 Group of Educational Research in Health Sciences, Department of Experimental and Health Sciences, Universitat Pompeu Fabra, Barcelona, Spain; 3 Hospital del Mar Institute of Medical Research (IMIM), Universitat Autónoma de Barcelona, CIBER de Epidemiología y Salud Pública (CIBERESP), Barcelona, Spain; Universidade do Porto Instituto de Biologia Molecular e Celular, PORTUGAL

## Abstract

The increasing perception that public communication in science and technology is an important tool to create a knowledge society is encouraging numerous public engagement activities. However, too little is known about scientists’ opinions of and attitudes towards the public with whom they interact during these activities, especially in southern European countries such as Spain. If we want to establish an effective dialogue between science and society, we need to be aware of the opinions and perceptions that both parties have of each other. In this study, we address this issue by focusing on 1022 responses to a survey conducted among scientists in Spain to discover their views of the public, and we then compare these responses with data from other national surveys on the public’s understanding of science. The results show that approximately 75% of Spanish scientists think that the general public has a serious lack of knowledge and understanding of scientific reasoning, although scientists do recognize that science interests the public (73%). Scientists believe that the public values the scientific profession to a lesser extent than suggested by public surveys: on a scale of 1–5, survey respondents rate their valuation of the scientific profession at 4.22, whereas scientists rate the public's valuation of the profession at 3.12, on average. Significant differences were detected between scientists’ perceptions of how citizens are informed about science and what citizens report in surveys. The challenge for the future is to narrow this gap in order to help scientists gain a better understanding of the public and their interests and to make public engagement activities more effective.

## Introduction

Scientific innovations are deeply embedded in social life: in the economy, in policy choices, and in how people care for themselves and how they use environmental resources. It is primarily for this reason that public communication in science and technology is increasingly seen as an important tool for creating a knowledge society. Indeed, in recent years, great importance has been placed in Europe on outreach and science dissemination activities [1, 2, 3], and most strategic documents describing research and development of the European Union and its member countries (including Spain) highlight the need to establish a dialogue between citizens and scientists [3, 4]. Over the last two decades, as interest in involving the public in research and innovation processes has increased, so has concern that these processes be implemented responsibly. This movement of responsibility, labeled responsible research and innovation (RRI), has been defined by Stilgoe et al. (2013) as “taking care of the future through collective stewardship of science and innovation in the present” [5].

Public communication of science tends to adopt different approaches that can be grouped into three models: dissemination model, dialogue model and conversation model [6]. The last model can be understood as "public engagement with science” and refers to a wide range of interactions that provide opportunities for mutual learning between scientists and members of the public [7]. Mutual learning refers not just to the acquisition of knowledge but also to increased familiarity with a wide range of perspectives, frameworks, and worldviews [8]. The challenge is therefore to encourage a deeper and more systematic engagement with civil society groups and the wider public.

For public engagement to make a difference, it must become part of the routine practice of this new concept of responsible science [7]. Despite the fact that calls for funding research (both from Europe and the Spanish government) increasingly include communication as a key issue, researchers’ involvement in such activities remains voluntary [9]. Moreover, most researchers do not receive specific training in these areas either as undergraduates or during their subsequent training as scientists (master's, doctorate, etc.) [10,11] Therefore, most researchers do not have specific consistent knowledge about effective strategies of scientific communication or about studies of social perceptions of science [9]. Therefore, their intuition, innate capacity for such tasks, or experience will determine their ability to participate in these activities. A substantial number of changes in Spain’s approach towards integrating science and society have occurred in the past few years. For example, public organizations in Spain now more often target public engagement. However, the inclusion of citizens in science and technology is not as developed as in some other European countries [12]. Public engagement is not an evaluation element, citizens are involved in research and innovation to a minor extent, and the organizational landscape enabling engagement of citizens is not well developed [12]. Nevertheless, the tendency is to align Spain’s strategy with the European guidelines.

The European Union strategy is clearly to move towards a deeper inclusion of society in the scientific process. In this context, public engagement activities are key, as is the willingness of scientists to take part. If the scientific community wants to establish an effective dialogue between science and society, it is important to be aware of the opinions and perceptions that both parties have of each other.

In this study, we address the issue of mutual opinions and perceptions by focusing on Spanish scientists’ views of the public and their role in public engagement activities. We compare these views to the results of previous national surveys on the public’s understanding of science from Spain and from Europe. We use the term “scientist” to refer to the broad range of individuals from across scientific, medical and engineering fields who are working in research and employed by universities or research institutions.

### What motivates scientists to be involved in science communication activities?

Unfortunately, there is scant literature that specifically analyzes Spanish scientists’ motivation to participate in science communication activities. Martin Sempere et al. studied this issue among scientists based in Madrid. The authors concluded that scientists are motivated by a wish to improve public interest and enthusiasm for science, the public’s scientific culture, and public awareness and appreciation of science and scientists [13]. However, scientists may also be motivated by a sense of duty, especially in the case of senior researchers [14]. Other studies suggest that the motivation is especially strong in the case of health science researchers, as they seek to promote medical science within the public sphere [15].

Dunwoody et al. and Besley et al. studied American scientists as public communicators. The primary motivation that scientists became involved in engagement activities were socials norms, a personal commitment to the public, good feelings of personal efficacy and professional obligation, and a desire to contribute to the public debate [16,17]. These findings indicate that researchers strongly believe that they should have a role in public debates. Bentley et al. studied the situation with climate change scientists and reported similar results especially when a substantial disjoint may exist between scientific findings and the impact they have on the public [18]. Nonetheless, Besley et al. showed that scientists from the United Kingdom specifically view policymakers as the most important group to engage with, rather than the general public [19].

De Boer et al. also reported that Irish food safety experts have little confidence in the public’s understanding of and ability to deal with scientific information and practices [20]. Other studies suggest that scientists engage with the public without reflecting on their own status as part of the public and act in accordance with their own linguistic and social domain [21]. This context leads researchers to favor the deficit model: one-way communication activities designed to educate the public and defend science from misinformation [14]. In contrast, scientists least prioritize communication that seeks to build trust and make the information imparted resonate with the public [22].

Collins et al. studied scientists’ usage of social media (Twitter, Facebook, LinkedIn or blogs) and concluded that researchers perceive numerous potential advantages to using social media in the workplace [23]. However, most scientists consider social media to be useful to communicate with other scientists rather than suitable for science communication to the general public [23].

Torres Albero et al. studied the Spanish situation and concluded that a contrast exists between researchers' desire to disseminate knowledge and the limitations derived from a low degree of interest in science in broad sectors of the Spanish society, together with professional promotion policies that do not give priority to science communication activities. The authors coined the term “trapped in a golden cage” to characterize the situation of scientists in Spain, who see no need for public engagement because they perceive little public demand for it. This situation led Torres Albero et al. to conclude that Spanish scientists are “trapped between the need to engage in dissemination activities from a moral standpoint and a social and professional environment (lack of time and of academic recognition) that is hardly conducive to these activities” [24].

### Why do scientists need to understand public perceptions?

As seen above, the traditional one-way approach to the science-society relationship still predominates in communication and outreach scenarios. However, many members of the public already understand basic scientific facts and concepts and therefore resist the presumption that they are not able to understand. Thus, “scientific education” alone may be insufficient for such citizens [25].

Sociodemographic factors such as gender, social background or ideology likely play a key role in how scientists view different dimensions of the public sphere [19]. These factors and others may also impact how scientists interact with the public and what they want to achieve by participating in these kinds of interactions [19, 26].

Some works reveal that scientists’ perceptions of the public vary according to the scientists’ experience [17, 19], field of research [27] and funding [28]. Greater experience in public engagement activities is associated with a better opinion of the public and with a more open-minded stance regarding how communication processes should be structured (e.g., dialogue or two-way communication rather than the idea of merely filling a knowledge vacuum) [19]. This range of perceptions entails that the term “public” is constructed in many different ways; indeed, it is important to consider “multiple publics.”

Some studies show that when scientists have closer contact with the lay public, they acquire a more nuanced view of this particular public and of the corresponding process of communication [25]. Torres-Albero et al. described the average profile of Spanish scientists who are involved in disseminating scientific findings as males who are over the age of 40 and working in the highest professional category. Scientists who are not involved in public engagement activities, however, were also predominantly male but older [24]. We have to take into account that this study was carried out in 2011 and that the profile of the typical scientist who participates in science communication in Spain may have changed since then. For example, early career researchers may have a key role in science communication as Donkor et al. reported in their studies with climate researchers from the United Kingdom and South Africa [29]. However, as far we know, there are no more recent studies addressing this issue in Spain.

### Public surveys about science and technology

Several countries have launched studies to investigate the characteristics of the relation between science and society so that appropriate strategies for improving the effectiveness of scientific dissemination can be designed. One such example is the Pew Research Center report [30], which focuses on a comparison of the views of the general public and scientists from the American Association for the Advancement of Science (AAAS). Another prominent example is the Science and Engineering Indicators report [31], last published in 2018 by the National Science Board of the United States; this biennial report provides a broad base of quantitative information about science, engineering, and technology, including public attitudes and understanding. In Europe, the European Commission regularly carries out opinion polls regarding science and technology [32,33] and on specific topic such as biotechnologies [34] or climate change [35]. In Spain, every two years, the Spanish Foundation for Science and Technology (FECYT) analyzes the relationships between science, technology and society through a survey [36].

In these studies, the survey questions are usually designed following six key indicators included in the three main dimensions of scientific literacy described by John D. Miller in 1998 [37]: information, interest, knowledge, understanding, opinions and attitudes and confidence.

The biannual surveys of FECYT provide data on the public understanding of science in Spain [36] based on these indicators. However, there are no data on scientists’ understanding of the perceptions of the public or of civil society organizations regarding scientists’ role in the research, development and innovation process or on Spanish scientists’ actual understanding of the public.

This gap supports the need for exploratory research to better understand the interaction between scientists and the public. If we want to promote public engagement and science dissemination, we need to understand researchers’ views, opinions and attitudes towards the public. Therefore, the present study sought to answer the following research questions:

RQ1. What are the opinions and attitudes of Spanish scientists towards the public?
RQ2. What are the opinions and attitudes of Spanish scientists towards public engagement with science?
RQ3. Are Spanish scientists’ views in line with data available in Spain on the public’s understanding of science?

## Materials and methods

The University Pompeu Fabra granted permission for this research (reference number 666004). All the participants of this research were informed about the study and asked to sign a written consent. All of them were free to answer each one of the questions as well as to stop participating at any time.

The population studied consisted of researchers living and working in Spain participating in research and development projects funded by the Spanish Ministry of Science, Innovation and Universities during 2013 and 2014. Accordingly, we can ensure that people participating in the study have lived and worked in Spain for a certain period of time and that they have been involved in a research project funded by the Spanish government. Henceforth, we will call this population "Spanish scientists."

The study is focused on Spain due to the need for a better understanding of the reality in Spain, a country where scientific communication is very active but that nonetheless remains largely absent from the international literature on the topic.

To answer our research questions, the study was carried out in two phases: a first exploratory phase in which 14 semistructured interviews were carried out with Spanish researchers to determine the scope of study and a second phase based on an online questionnaire completed by Spanish researchers.

The study was conducted as a collaborative process between the research team (the authors and 2 masters’ students who only collaborate in data collection) and a working group composed of 14 representatives of Spanish Scientific Culture Units, which are structures formally recognized by the Spanish government and based at universities and research centers. These structures act as intermediaries between their host institutions and citizens with the main aim of promoting scientific, technological and innovation culture though different types of activities: scientific communication, outreach, training, etc. These units are key agents in the dissemination of science and innovation in Spain and provide a crucial service for improving and contributing to the training, culture and scientific knowledge of citizens [38]. Table 1 summarizes the institutions that selected a representative as part of this working group:

**Table 1:** Working group institutions.

University or research center	City	Autonomous community
University of Seville	Seville	Andalusia
University of Zaragoza	Zaragoza	Aragon
National Research Centre on Human Evolution (CENIEH)	Burgos	Castile and León
University Jaume I	Castellón de la Plana	Community of Valencia
Polytechnic University of Madrid	Madrid	Madrid
University Carlos III	Madrid	Madrid
University of Cordoba	Córdoba	Andalusia
International University of La Rioja	Logroño	La Rioja
Seneca Foundation	Murcia	Murcia
University of the Basque Country	Leioa-Bizkaia	Basque Country
AZTI-Tecnalia	Pasaia, Guipuzkoa	Basque Country
Institute for Bioengineering of Catalonia	Barcelona	Catalonia
Open University of Catalonia	Barcelona	Catalonia
Pompeu Fabra University	Barcelona	Catalonia

The working group collaborated with the research team throughout the study in several key points: definition of the study dimensions, revision of the script of the semistructured interviews, determining the survey design and the sample selection. We also relied on the experience of the working group during the discussion of the results to ensure the reliability of the interpretations. The units of scientific culture were selected taking into account the geographical distribution of the researchers in the country.

### Scope of study

To map the different views, define the scope of the study, and devise the specific questions to be included in the online survey, we conducted 14 semistructured interviews. The main objective of these interviews was the qualitative exploration of scientists’ views on different subjects related to our research questions, such as the public interest in science and technology, the public perception of scientists’ profession, the public image of science and technology, the scientific cultural level of citizens, how the public is informed about science, and their role in public engagement activities.

The specific script of the semistructured interview was prepared following the guidelines of Martín Izard [39] and was devised jointly by the abovementioned working group and the research team. Before participating, representatives were informed about the study and asked to sign a written consent form. The research team developed a semi-structured interview protocol and two interviewers conducted face to face or Skype interviews. All the interviews were recorded, transcribed immediately, and shared with the research team. For reasons of privacy protection, the names of the representatives and the organizations have been anonymized. Feedback was also obtained from the working group associated with the project. One researcher from each unit’s institution was interviewed during the spring of 2016.

We interviewed seven men and seven women. In choosing the sample, we took into account research experience (over or under 10 years working in research), the degree of involvement in public engagement activities (participating in more than or fewer than 3 activities per year) and the research field. Each member of the Spanish Scientific Culture Units included in our working group suggested one researcher from their university or research center according to the selection criteria noted above. Finally, we included researchers from the fields of biology, engineering, paleontology, veterinary science, physics, mathematics and chemistry to gather a diversity of views. Researchers from social sciences and humanities were not included, but the research team decided that the variety of disciplines was wide enough to perform this exploratory study.

Based on these semistructured interviews and our research questions, the study explores the following themes:

- *Scientists’ opinions of and attitudes towards the public*: How scientists perceive public interest in science and technology, how scientists view public understanding of science and technology, and the public image and perception of scientists and the profession. In this section, we also analyze how scientists believe the public is informed about science and technology-related issues (RQ1 and 2).
- *Scientists’ opinions of and attitudes towards their role in public engagement activities*: How scientists should be involved in science dissemination (RQ3).

### Survey design

The specific questions of the survey were developed in close consultation with the working group. The survey design incorporated the key indicators and certain questions used in surveys on public understanding of science, such as those carried out every two years by the FECYT [36] or the Science and Engineering Indicators report [31], published by the National Science Foundation of the United States, in order to be able to compare the answers. The survey also included new questions to provide a broader knowledge as well as certain sociodemographic questions.

The exact wording and complete questionnaire are available in S1 Questionnaire. Specifically, questions 2, 3, 5, 6 and 8 of the questionnaire are inspired by the public perception surveys of the FECYT [36]. Of these questions, questions 3, 5 and 6 are the same questions that the FECYT used in their survey. Of course, the wording has been changed since the FECYT study focuses on the responses of society, whereas our study focuses on the responses of researchers. Question 4 is inspired by a question from the Science and Engineering Indicators report. Similarly, readjustments were made in the wording due to the difference in the target audience in the two studies. Questions 1, 7, 9 and 10 were defined by the research group taking into account the scope of the study and researchers’ responses in the exploratory interviews.

The final online questionnaire consisted of nine questions about researchers' perceptions of society, seven of which were multiple-choice questions in which the researchers had to select the option that best suited their opinion. In the other two questions, the scientists were asked to indicate their degree of agreement based on a Likert scale. In addition, a tenth multiple-choice question asked the researchers which professionals should be involved in science dissemination. The questionnaire was accompanied by an informative e-mail specifying the purpose of the study and the use that was to be made with the data that the participants provided. Respondents were free to choose whether or not to answer any particular question. Before submitting their answers, respondents had to check that they have received informed consent. The average survey duration was approximately 15 minutes.

### Survey sampling

According to data from the Statistics Institute of the Spanish Government [40], the number of Spanish researchers dedicated to research and development was 122,235, a figure representing the maximum size of the study population. We did not take into account scientists working in commercial companies, who, according to the Statistics Institute, number approximately 45,000 people in Spain [36]. Thus, the study inferences concern scientists working in public universities or research centers.

Adhering to the tailored design method [41], each member of the working group made three contacts with members of the sample of their institution over approximately four weeks and sent e-mail reminders once responses from the first invited contact had been received. Finally, the online survey was distributed to 5554 scientists from the working group institutions. The response rate was 22%, resulting in a final data set of 1022 scientists (sampling margin of error of approximately 3% and a 95% confidence level). The response rate to online questionnaires is usually relatively low, but this response rate is consistent with those reported for other online surveys of expert communities (e.g., [22]). Moreover, this is a descriptive study that does not seek to analyze precise differences between participants but, rather, to provide a group description. The online survey was sent during the autumn of 2016.

The data were weighted to ensure that the demographics of the sample reflected the underlying population of scientists; that is, we have taken into account factors such as gender (60% male and 40% female), age, research experience, and field of study (see Table 2). The field division used is the one provided by the Spanish Ministry of Science, Innovation and Universities.

**Table 2:** Age, research experience and field of study.

**Age group**	**N**	**% of the sample**
Under 24 years old	42	4
25 to 34 years old	257	25
35 to 44 years old	274	27
45 to 54 years	280	27
55 to 64 years old	139	14
Over 65 years old	30	3
**Level of experience**	**N**	**% of the sample**
1 to 10 years	335	33
11 to 20 years	268	26
21 to 30 years	279	27
31 to 40 years	112	11
Over 41 years	28	3
**Field of study**	**N**	**% of the sample**
Exact and natural sciences	374	37
Engineering and technology	241	24
Medical sciences	228	22
Agricultural sciences	37	4
Social sciences	108	11
Humanities	34	3

To study the relationships between the different variables, we performed Pearson's chi-squared test with the statistical software Statistical Package for the Social Sciences (SPSS), version 23.

## Results

### Scientists’ views of public interest in science and technology

As we noted in previous sections, the question regarding public interest in science and technology was defined by the research team taking into account the researchers’ responses in the exploratory interviews and questions on the same topic proposed in the FECYT study [36].

During these interviews, the answers included in Table 3 were mentioned enough times (in more than 7 interviews) to be considered as an item for study. The wording of answer 4"No, Spanish society has no interest in knowing more about science and technology" derives from the idea that, usually, when one is interested in something, one wants to "know more" about the subject. This same action of "wanting to know more" appears in the FECYT studies noted above related to society's interest in science and technology issues [36].

**Table 3:** Scientists' views of public interest in science and technology (total and according to gender).

Do you consider that Spanish society is interested in knowing more about scientific and technological issues?			
	Female	Male	Total
Yes, Spanish society is interested in science and technology	11.5%	15.1%	13.7%
Yes, Spanish society is interested only in health, food and applied science	35.6%	28.3%	31.2%
Yes, Spanish society is interested in science and technology but has a lack of understanding	29.7%	27.0%	28.1%
No, Spanish society has no interest in knowing more about science and technology	23.1%	29.5%	26.9%

Approximately 73% of the scientists surveyed considered that Spanish society is interested in science and technology issues (Table 3). However, more than one-third think considered the interest is only related to applied science or specific topics, such as health or nutrition. Women support this view more strongly than men (36% vs. 28%, p = 0.001). Additionally, 28% of the sample asserted that despite being interested in this field, the public has a lack of understanding of science and technology. The percentage of women supporting this view is also slightly higher than that of men (30% vs. 27%, p = 0.001).

The view that the public has no interest in science and technology was slightly more common in men than in women (30% vs. 23%, p = 0.001). There were also differences by field of study: those working in the engineering and technology fields (33%) were less confident about public interest in science and technology than were scientists in the other groups (p = 0.001). Scientists working in the humanities showed more positive views (38%), especially regarding public interest in applied science (p = 0.001).

### Scientists’ views about the public image of science and technology as a priority

One of the most classic indicators of the degree of commitment of citizens to science is whether they consider public spending in this area a priority. The answers of scientists to the corresponding question are listed in Table 4.

**Table 4:** Scientists' views on the public prioritization of public investment in science and technology.

Do you think that public funding for science and technology is a priority for Spanish society?	
Yes, Spanish society considers public investment in science and technology to be a priority	8%
Yes, but public investment is accepted more in applied science than in basic science	35%
No, Spanish society does not consider public investment in science and technology to be a priority	57%

Of the sample, 57% agreed that Spanish society does not consider public investment in science and technology to be a priority. This view was more commonly supported by male than by female respondents (61% vs. 53%, p = 0.002). Over a third of the respondents indicated that society accepts this type of investment more readily in applied science than in basic science. In this case, female respondents were slightly more positive than males (41% vs. 31%, p = 0.002).

### Scientists’ views of the public knowledge and understanding of science and technology issues

As shown in Table 5, with respect to the cultural level of Spanish society in terms of science and technology compared with that in other European countries, most of the sample (75%) reported that it is low or very low, with only 1% viewing it as high or very high. There were no statistically significant differences by gender, experience or research fields.

**Table 5:** Scientists' views of the level of scientific culture in Spain.

What would you say is the level of scientific culture in Spain compared to other countries in the European Union?	
Very low	17.5%
Low	57.8%
Normal	23.3%
High	1.3%
Very high	0.1%

Table 6 summarizes scientists’ answers to a question on the capacity of citizens to correctly choose the best way to test the effectiveness of a treatment against high blood pressure. Most of the scientists surveyed think that a quarter of Spanish society could answer this question correctly, whereas 21% of them consider that Spanish society is not capable of answering this question correctly. This opinion was shared more widely among men than women (24% vs. 17%, p = 0.037). As the age and research experience of the scientists surveyed increased, the confidence in Spanish society’s ability to respond to questions of this kind decreased (16%, 17%, 26%, 27% and 36%, p = 0.019).

**Table 6:** Scientists' views of public understanding of a scientific process (role of a clinical trial).

**Some questionnaires regarding public perception of science include questions such as these:**	
Imagine that two scientists want to know whether a given substance is effective against hypertension: • Scientist A proposes studying 1000 people with hypertension by giving the substance to all of them and observing how many people experience a decrease in their blood pressure. • Scientist B proposes studying 1000 people with hypertension but giving the substance to only 500 people (leaving the other 500 to follow their usual treatment) and observing how many individuals in each group experience a decrease in their blood pressure. Which of the two scientists proposes the best way to test the drug?	
**Do you consider that society is able to answer this question correctly?**	
Less than a quarter of Spanish society can answer this question correctly.	21%
A quarter of Spanish society could answer this question correctly.	55%
Half of Spanish society could answer this question correctly.	22%
More than half of Spanish society could answer this question correctly.	2%

### Scientists’ views of the public perception of scientists and their profession

**Fig 1:**
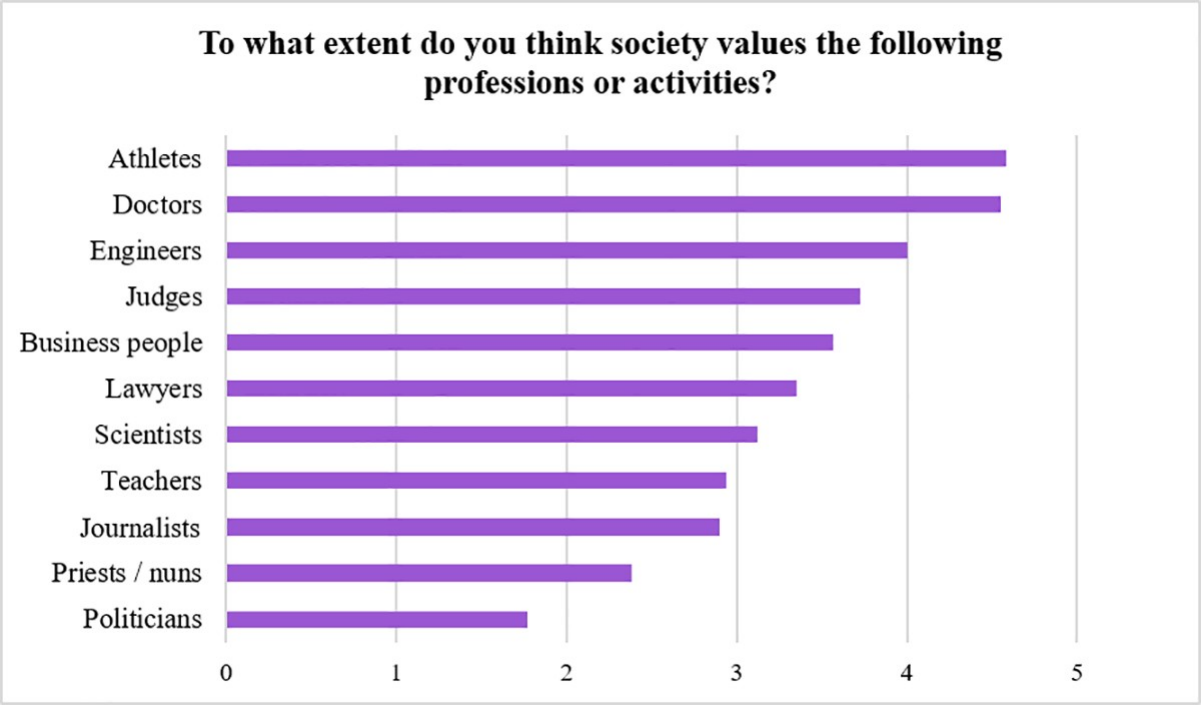
Scientists’ views of public valuing of professions.

We replicated a question devised by the FECYT by asking scientists to indicate how Spanish society values 11 different professions on a scale of 1 (poorly valued) to 5 (highly valued). As we can see in Fig 1, Spanish scientists believe that athletes are the professional group valued the highest by the public and politicians the group valued the least. Spanish scientists also consider that scientists are less highly valued than doctors, engineers, judges, businesspeople and lawyers, with scientists ranked in seventh position. There were no statistically significant differences by age, gender, experience or research fields.

To gain further knowledge about scientists’ perceptions of the public recognition of their profession, we asked them to select the opinions that were most closely aligned with their own opinions from a list of options derived from FECYT surveys. The exact wording and responses are shown in Table 7. Most answers reflect scientists’ perception that their job is not valued enough by the public.

**Table 7:** Scientists’ views of the public perception of issues related to scientists.

Mark the statement that best fits your opinion	
Spanish society feels that research is a male-oriented profession.	14%
Spanish society believes that research is an underpaid profession.	37%
Spanish society sees research as a profession lacking in job stability.	46%
Research is useful to Spanish society, but the public does not consider it to be a priority.	88%
Spanish society does not see research as a profession that is useful to society.	18%

Other facets of the same perception can be seen in Table 8. Notably, only 3% of respondents considered that “society has a real image of the actual work of the researcher,” while the option “society has a stereotypical image of the researcher” was chosen by 79% of respondents. The latter view was held by 85% of researchers from the fields of exact and natural sciences, by 80% of those in engineering and technology, but only by 31% of scientists from the humanities.

**Table 8:** Scientists’ views of the public perception of scientists.

Mark the statement that best fits your opinion	
Spanish society respects and values scientists as pillars of modern society.	12%
Spanish society considers that scientists are a social point of reference, but only in their respective fields.	60%
Spanish society has a stereotypical image of researchers as mad scientists, eccentric, very clever, capable of solving anything but disconnected from real life.	79%
Spanish society has a realistic perception of the actual work of researchers’ routines, i.e., reading and writing scientific articles, looking for funding, etc.	3%

### Scientists’ views of how the public is informed about science and technology

**Fig 2:**
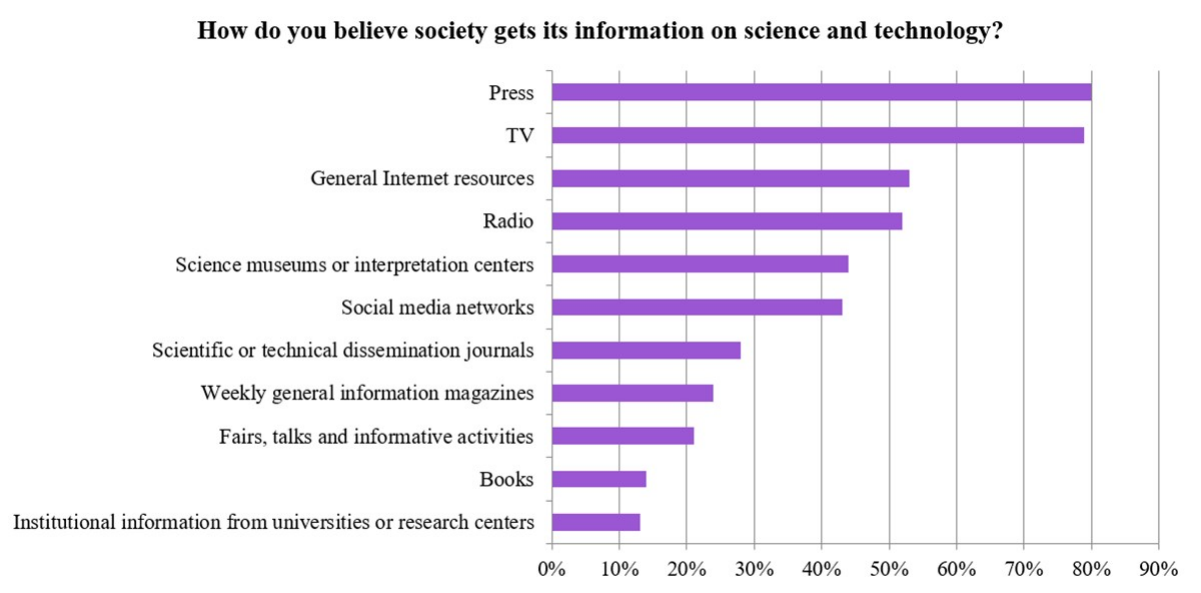
Scientists’ views of how the public is informed about science and technology.

Asked about “how society is informed about science and technology,” the majority of scientists selected the press and TV (see Fig 2).

Less experienced scientists (fewer than 20 years in research) and the most experienced scientists (more than 40 years in research) tended to consider TV as a less important channel for information. If scientists’ views are correct on the less commonly used means of information, there is a high potential to increase the use of informative activities, books, and institutional channels.

### Scientists’ views of their role in public engagement activities

We also assessed who scientists thought should be involved in science communication and public engagement activities. They could choose more than one option from the list shown in Table 9. Their answers suggest there is broad potential to strengthen collaborations among researchers and communication specialists. There were no statistically significant differences by gender, age, experience or research fields.

**Table 9:** Scientists’ views of who has to communicate or disseminate science and technology.

In your opinion, who should communicate or disseminate science and technology?	
Research staff	69%
Specialized communication staff linked to the research center or university	87%
Journalists or specialized communicators	74%
No one	1%

**Fig 3:**
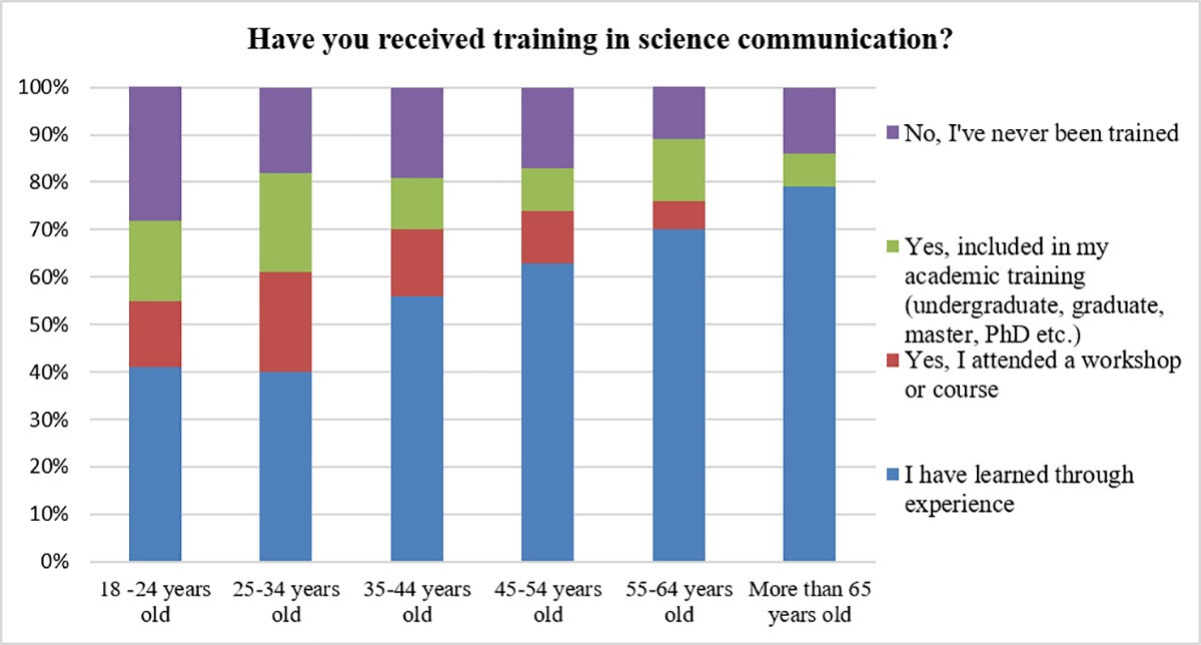
Training in scientific communication.

Moreover, there was a clear inverse relationship between scientists’ age and having received some training in science communication (Fig 3) (p for trend <0.001).

## Discussion

The results show that Spanish scientists think that the general public has a serious lack of knowledge and understanding of scientific reasoning, although researchers recognize that science interests the public. Researchers also believe that the public’s valuation of the scientific profession is low and that the press is the most widely used source of information. Spanish scientists consider communication and dissemination of science to be a shared responsibility among research staff, institutional communicators and scientific journalists. There is broad potential to strengthen collaborations among researchers and communication specialists, for example, by funding programs for science communication activities, through awards for science journalism, formal recognition of communication activities in the scientists’ curriculum’, etc. The clear increase in training in science communication of younger researchers further supports the possibilities of current efforts to educate researchers in science communication.

**Fig 4:**
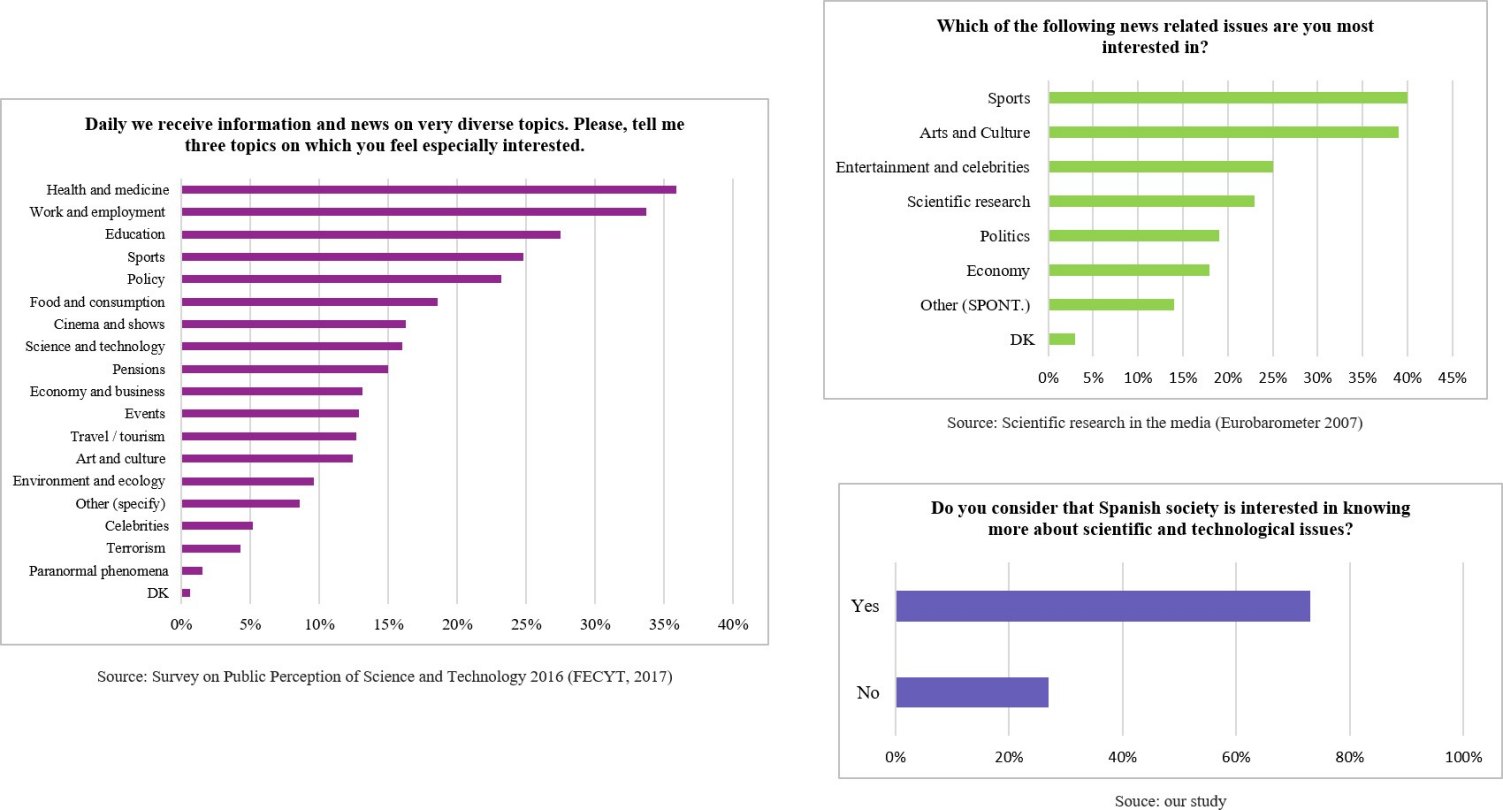
The perceived level of interest in science and technology by society in general (FECYT and Eurobarometer data) and scientists (our data).

As we reported in previous sections, Spanish scientists have a quite positive perception (73%) of the level of public interest in science and technology. The last survey of the FECYT [36] shows a slight increase in the degree of interest in science of 7% from the 2008 survey), although interest is still low. Similar data were also reported by the Eurobarometer survey “Scientific research in the media”: only 23% of the surveyed Spaniards showed interest in scientific research, placing this topic in fourth place, while in other countries such as Sweden, Greece, France, Belgium, Cyprus and Luxembourg, it is ranked in first place [42]. Thus, Spanish scientists have a more positive perception of the level of public interest than those observed in other public surveys (see Fig 4).

**Fig 5:**
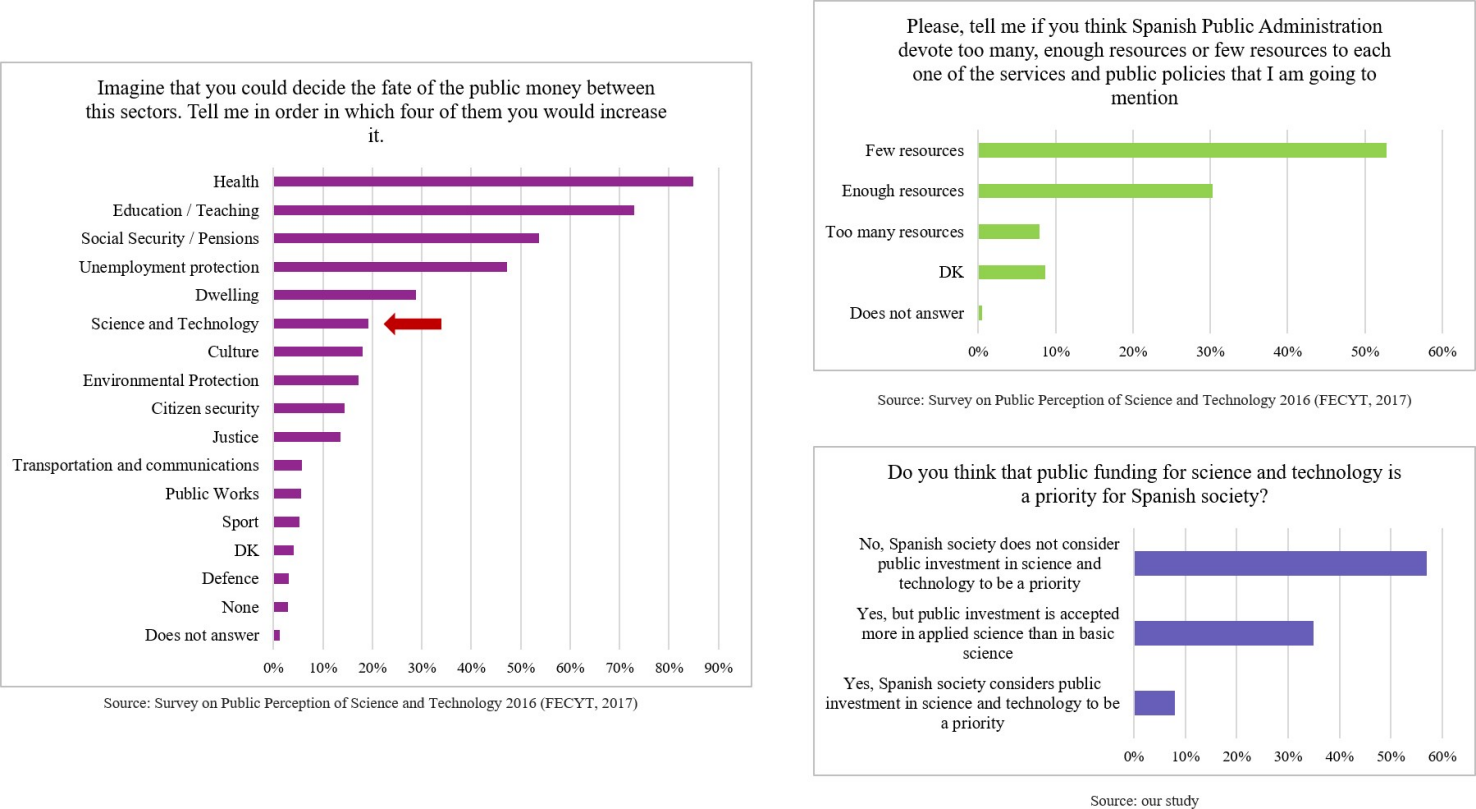
Prioritization of public spending in science and technology by society in general (FECYT data) and scientists (our data).

The last FECYT report concluded that the areas in which citizens would increase public spending were mainly health and education, while science and technology were ranked in sixth place [36]. This report also shows that 53% of the surveyed Spaniards believe that the level of commitment of the Spanish government to research is insufficient. However, other factors to be taken into account are the economic situation of Spain in the last few years and how the recession influenced priorities in public spending to cover other social needs. As we have seen, 57% of the surveyed scientists agreed that Spanish society does not consider public investment in science and technology to be a priority. With this in mind, our results suggest that scientists’ views of this issue are more negative than those of the rest of society (see Fig 5).

The view of the public as uninterested and incapable of understanding scientific research was also reported by Besley et al. [19]. Our impression is that this perception leads scientists to think that society does not have the necessary skills to understand how important public investment in science and technology truly is.

We note a concept that commonly appears in the researchers' responses: scientists tend to consider the public to be much more knowledgeable and sensitive in relation to scientific issues that affect them directly.

**Fig 6:**
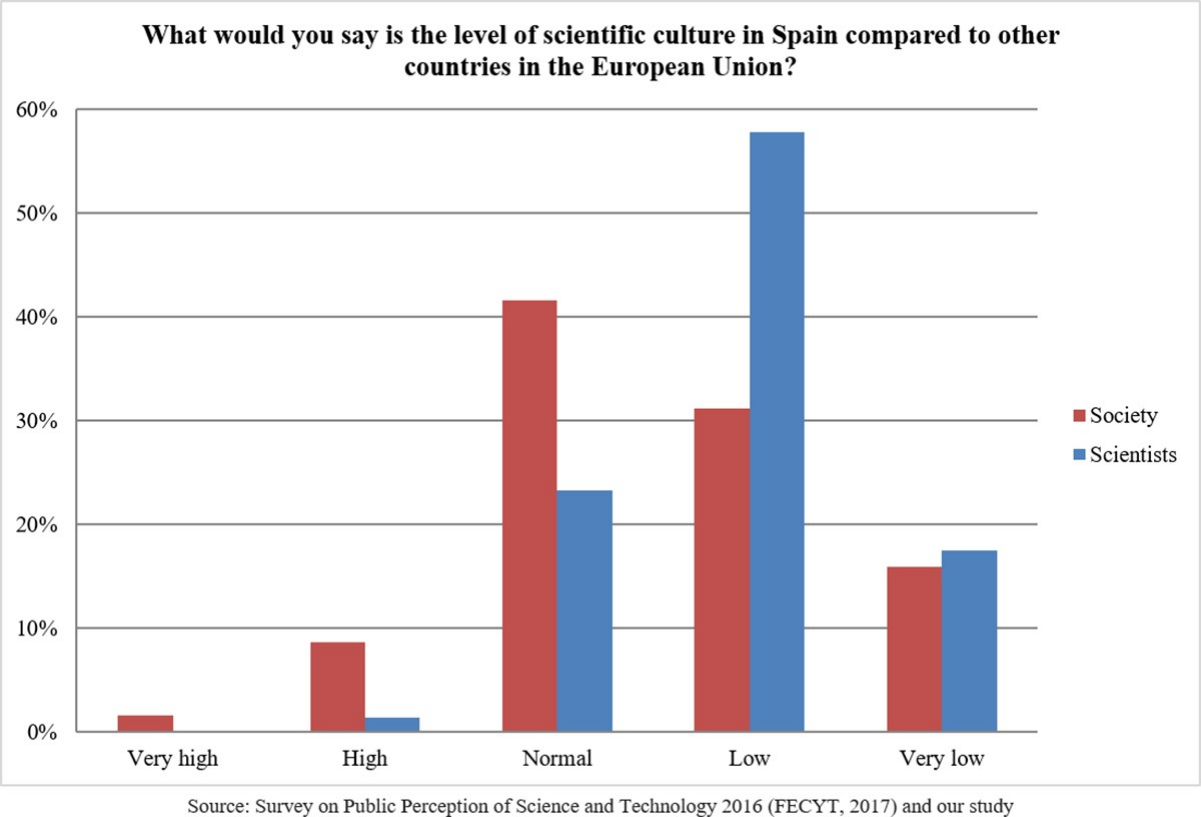
The perceived level of scientific culture in Spain compared to other European countries by society in general (FECYT data) and scientists (our data).

In general, Spanish scientists think that the public has a lack of scientific culture. Comparing these results with those provided by the FECYT [36] shows that the view of scientists is clearly less positive than the public’s self-view (see Fig 6). As we can see in Fig 6, the majority of the surveyed scientists agreed that Spanish society has a low (option chosen by 58% of scientists) or very low (18%) level of scientific culture. In contrast, the results of the FECYT study [36] show that most members of the Spanish society consider their own level of scientific culture to be normal (42%), or lower (31%) than the level of other European countries.

Some studies of scientists’ views of the public showed that the most widespread view among researchers is that the main barrier to a greater understanding of science among the public is a lack of science education [19]. This is also in line with the traditional deficit model of science communication that sees scientific illiteracy as the key problem [25].

In our study, some scientists pointed out that the lack of knowledge of science does not contradict or interfere with public interest in knowing more about science and technology. This position goes beyond the deficit model, which attributes public skepticism or hostility to science and technology to a lack of understanding resulting from a lack of information.

Understanding how clinical trials work implies a knowledge of the nature of science and key elements such as the need for control or replication to validate a study. However, the results from the present study suggest that scientists, especially men, feel that most of the Spanish population does not have the capacity to understand specific scientific knowledge, such as clinical trials. The Science and Engineering Indicators report published by the National Science Board [31] offers results regarding the same question on public understanding of scientific experiments similar to the views of Spanish scientists.

The exact same question was not answered by Spanish society, but the last FECYT survey [36] included a different question regarding clinical trials with several answers to choose from, where the majority of the population did not know the scientific procedures, which is also in line with the scientists’ predictions in our survey. Of course, more studies are needed on the degree of understanding of Spanish society on different scientific concepts to understand the reality of the situation. However, the main finding is that Spanish scientists consider that the majority of Spanish society is not able to understand key aspects of the scientific process.

Older researchers, who are not as involved in public engagement activities as younger ones, have a more traditional view of the public [25] based on the deficit model. Such results are consistent with the typical Spanish scientists involved in dissemination activities described by Torres-Albero et al [24]. Moreover, our study also shows that younger scientists tend to have more formal training in science communication than their older colleagues. Therefore, training in communication or public engagement could be beneficial for improving scientists’ perceptions of the public since it could ensure a more realistic understanding of who the public are and what the public want.

**Fig 7:**
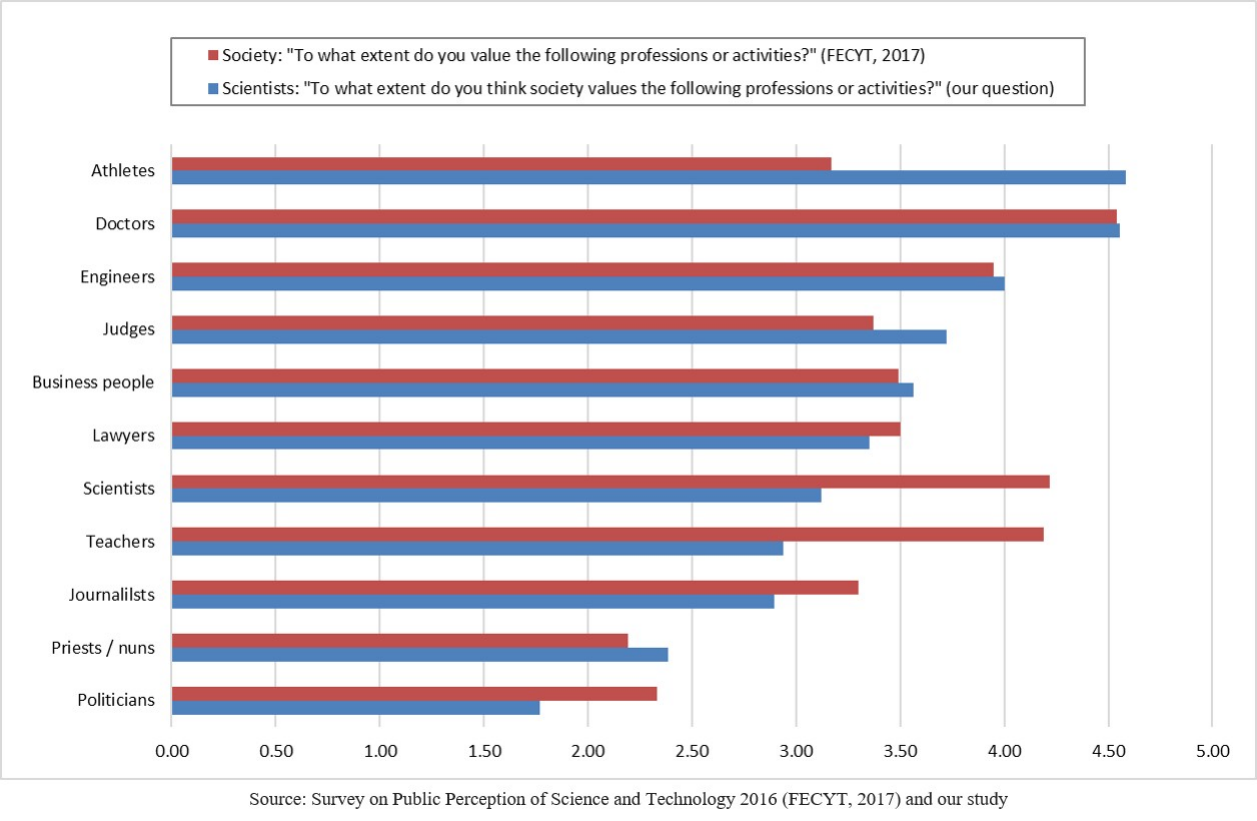
The perceived level of public recognition of professions by society in general (FECYT 2016 data) and by scientists (our data).

Comparing our results with those from the last FECYT report (see Fig 7), we can see that Spanish society values scientific professions more than researchers believe [36].

Moreover, studies on changes in the public recognition of professions indicate a growing level of recognition of doctors, scientists and teachers and, most recently, of engineers [36]. Thus, Spanish society ranks all professions related to science and technology (doctors, scientists and engineers) among the most highly valued groups.

It appears that in Spanish society, the scientific profession enjoys a high level of recognition, yet some studies suggest that scientists do not believe so [39, 9]. Other studies related scientists’ view to a concern about being identified as a potential target of criticism; they also related such a view with the belief that the public would misunderstand any attempt at communication and either make the scientists look bad or misuse their work [25].

These views could be related to those reported by scientists in our survey, where scientists consider that society does not have a true image of researchers because the public is not familiar with their profession. These views were especially supported by researchers in the field of exact and natural sciences, which is perhaps the field of study with the least amount of direct contact with society. Despite this perception, scientists also believe that society considers them as a point of reference in their respective fields.

**Fig 8:**
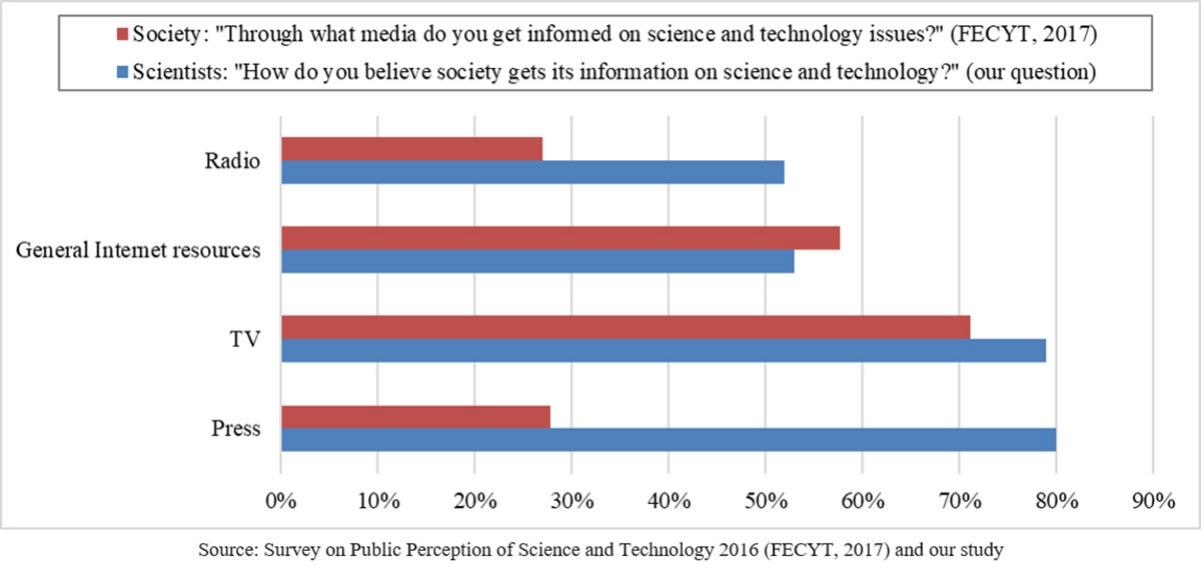
The perceived main sources of science and technology information by society (FECYT 2016 data) and by scientists (our data).

However, as we can see in Fig 8, the press is seen by scientists as the most widely used source of information, while considerably less than half of society actually considers this [36] to be the case. This perception could be due to the great importance that universities and research centers give to press clippings as a measure of their impact in mass media.

The data show that scientists consider that scientific communication is a shared responsibility among the institutional communication departments, journalists and researchers. A higher proportion of scientists in our survey considered that the communication and dissemination of science were the responsibility of other specialists rather than of researchers. However, some studies have observed that the public places a high value on the participation of scientists in this kind of activity [43]. We must also bear in mind that in Spain, participation in public engagement activities is virtually always voluntary and that scientists involved seldom receive formal recognition for this work. Our findings suggest that scientists’ efforts could be better recognized institutionally and academically, and perhaps also socially; this would not only legitimize public engagement efforts but also make them normative [44].

## Conclusions

Overall, our findings provide an overview of Spanish scientists’ opinions and attitudes towards citizen’s understanding of science and on scientists’ own role in public engagement activities. The findings thus enable us to identify the differences and to propose strategies to improve citizen participation in the research process.

### Opinions and attitudes of Spanish scientists towards the public

Many scientists in Spain still believe that the lack of public scientific knowledge is the key hindrance to society being involved in public engagement activities. This belief fits better with a one-way communication approach than with approaches focusing on mutual learning processes or public engagement activities.

However, it is the latter approach that is currently being promoted by many institutions and organizations worldwide, including Spain. Knowing what Spanish scientists think about the public is key to promoting rapprochement between the scientific community and society. On the one hand, we believe that it is necessary to increase researchers’ knowledge of public perception of science and technology. A good option to do this would be through training programs included in formal scientific education (undergraduate degrees, master's and doctoral programs).

On the other hand, if the science and technology system truly wants to encourage the involvement of researchers in public engagement activities in science, these types of actions should be formally recognized for career promotion.

### Opinions and attitudes of Spanish scientists towards public engagement in science

Scientists consider public engagement activities to be a shared responsibility among institutional communication departments, journalists and researchers. The advantage of this situation is that Spanish scientists have a willingness to participate in scientific communication activities. In addition, they consider that scientific communication is a multidisciplinary activity involving collaboration among different actors.

Despite this, we have seen that scientists’ confidence in public capacities decreased with increasing age. This finding is more related to the deficit model approach described above than to participatory activities such as public engagement. However, other studies have reported that a greater contact with science communication and experience in outreach activities improves views on public abilities and capacities [25]. Therefore, it is plausible that these findings are explained by a relative lack of experience in such activities by the oldest cohorts of scientists; this process also seems likely in countries other than Spain. Therefore, we believe that promoting mutual learning and public engagement activities by the scientific institutions and funding agencies is crucial to improve this situation.

We also observed that younger generations tended to receive more specialized training in scientific communication. Our hypothesis is that including formal training in science communication and public understanding of science during a scientist’s research career could foster a more realistic view of the public and help to boost public engagement. Obviously, more research must be carried out to better assess this issue.

### Differences between Spanish scientists’ views and public understanding of science

There are some differences between the perceptions of scientists analyzed here and the perceptions of the public collected in the biannual reports of the FECYT [36]. Specifically, these differences relate to the sources of information used by the public, the level of public recognition that science and researchers receive, the level of scientific education and the level of interest in science and technology.

Identifying these differences in perceptions is important to propose strategies that improve the relationship between the scientific community and society. For example, if scientists know what sources of information the public uses the most, they are probably more willing to participate in scientific communication activities that may have an impact on these channels. Similarly, awareness of the level of social valuation that their profession has can influence the scientists’ perception of the public. This may also favor the willingness among scientists to participate in public engagement activities.

The challenge for the future is to explore how to close such gaps in perceptions so that scientists can have a better understanding of the public and its interests and carry out public engagement activities efficiently. Through these kinds of activities, scientists can enter into discussions with a wide range of stakeholders, allowing questions and concerns to be better understood and addressed. By doing so, scientists can connect different points of view, change aspects of their work, and make it more relevant to society.

## Supporting information

S1 Questionnaire Exact wording and complete questionnaire. (PDF)
