# Supplementary material for: Scientists’ opinions and attitudes towards citizens’ understanding of science and their role in public engagement activities
Source: PLoS One. 2019 Nov 13;14(11):e0224262. doi: 10.1371/journal.pone.0224262 (PMC6853295; doi:10.1371/journal.pone.0224262)
Supplement: S1 Questionnaire — (PDF) [file pone.0224262.s001.pdf]

Scientists’ opinions and attitudes towards citizens’ understanding of science and their role in public engagement activities

This questionnaire is aimed at research staff participating in R&D&I projects funded by the Ministry of the Economy and Competitiveness (*Ministerio de Economía y Competitividad,* MINECO) in 2013 and 2014.

All the data gathered shall be treated as confidential and used in a research study on public perception undertaken by Pompeu Fabra University and funded by the FECYT (Spanish Foundation for Science and Technology).

1. Do you consider that Spanish society is interested in knowing more about scientific and technological issues?

1. *Tick one only.*

- Yes, Spanish society is interested in science and technology
- Yes, Spanish society is interested only in health, food and applied science
- Yes, Spanish society is interested in science and technology but has a lack of understanding
- No, Spanish society has no interest in knowing more about science and technology

2. Do you think that public funding for science and technology is a priority for Spanish society?

2. *Tick one only.*

- Yes, Spanish society considers public investment in science and technology to be a priority
- Yes, but public investment is accepted more in applied science than in basic science
- No, Spanish society does not consider public investment in science and technology to be a priority

3. What would you say is the level of scientific culture in Spain compared to other countries in the European Union?

3. *Tick one only.*

- Very low
- Low
- Normal
- High
- Very high

4. Some questionnaires regarding public perception of science include questions such as these:

Imagine that two scientists want to know if a given substance is effective against hypertension:

- Scientist A proposes studying 1000 people with hypertension by giving the substance to all of them and observing how many people experience a decrease in their blood pressure.
- Scientist B proposes studying 1000 people with hypertension but giving the substance to only 500 people (leaving the other 500 to follow their usual treatment), and observing how many individuals in each group experience a decrease in their blood pressure.

Which of the two suggestions is the best way of testing the drug?

4. ***Do you believe that society is capable of answering this question correctly?***

   Tick one only.

   - Less than a quarter of Spanish society cannot answer this question correctly.
   - A quarter of Spanish society could answer this question correctly.
   - A half of Spanish society could answer this question correctly.
   - More than a half of Spanish society could answer this question correctly.

5.To what extent do you think society values the following professions or activities?

5. *Tick one only per row: 1 poorly valued to 5 highly valued.*

	1	2	3	4	5
Doctors					
Scientists					
Engineers					
Judges					
Lawyers					
Athletes					
Journalists					
Business people					
Teachers					
Priests/nuns					
Politicians					

6. Mark the statement that best fits your opinion:

- [ ] Spanish society feels that research is a male-oriented profession.
- [ ] Spanish society believes that research is an underpaid profession.
- [ ] Spanish society sees research as a profession lacking in job stability.
- [ ] Research is useful to Spanish society, but the public do not consider it to be a priority.
- [ ] Spanish society does not see research as a profession that is useful to society.

7. Mark the statement that best fits your opinion:

- [ ] Spanish respects and values scientists as pillars of modern society.
- [ ] Spanish society considers that scientists are a social point of reference, but only in their respective fields.
- [ ] Spanish society has a stereotypical image of researchers as mad scientists, eccentric, very clever, capable of solving anything but disconnected from real life.
- [ ] Spanish society has a real image of the actual work of researchers’ routines, i.e. reading and writing scientific articles, looking for funding, etc.

8. How do you believe society gets its information on science and technology?

8. Select all the relevant options.

- [ ] Press
- [ ] Books
- [ ] Radio
- [ ] Scientific or technical dissemination journals
- [ ] Weekly general information magazines
- [ ] TV
- [ ] General Internet resources
- [ ] Science museums or interpretation centres
- [ ] Fairs, talks and informative activities
- [ ] Social media networks
- [ ] Institutional information from universities or research centres

9. In your opinion, who should communicate or disseminate science and technology?

9. Select all applicable options.

- ☐ Research staff
- ☐ Specialized communication staff linked to the research centre or university
- ☐ Journalists or specialized communicators
- ☐ No one

10. Have you received training in science communication?

10. Indicate all the options you agree with

Tick one only.

- ◯ Yes, included in my academic training (undergraduate, graduate, master, PhD etc.)
- ◯ Yes, I've attended a workshop or course
- ◯ I've learned through experience
- ◯ No, I've never been trained

Socio-demographic information

This information is anonymous and confidential, and shall only be used for the purposes of this research.

11. **Indicate your gender**

Tick one only.

- ◯ Female
- ◯ Male
- ◯ Other

12. **Indicate your age**

Tick one only.

- ◯ Under 24 years old
- ◯ 25-34 years old
- ◯ 35-44 years old
- ◯ 45-54 years old
- ◯ 55 - 64 years old
- ◯ Over 65 years old

13. **Indicate the number of years you have worked as a researcher**

Tick one only.

- 1 to 10 years
- 11 to 20 years
- 21 to 30 years
- 31 to 40 years
- Over 41 years

14. **Indicate your field of study**

Tick one only.

- Exact and natural sciences
- Engineering and technology
- Medical sciences
- Agricultural sciences
- Social sciences
- Humanities

15. **Indicate the Autonomous Community in which you work**

Tick one only.

- Andalusia
- Aragon
- Principality of Asturias
- Balearic Islands
- Canary Islands
- Cantabria
- Castile-La Mancha
- Castile and León
- Catalonia
- Community of Valencia
- Extremadura
- Galicia
- La Rioja
- Community of Madrid
- Chartered Community of Navarre
- Basque Country
- Region of Murcia
- Ceuta
- Melilla
